# Establishment of prognostic prediction model based on lipid metabolism related genes in esophageal squamous cell carcinoma by machine learning algorithms

**DOI:** 10.1186/s12876-026-04908-0

**Published:** 2026-05-12

**Authors:** Peng Wang, Zhenyuan Feng, Weigang Chen

**Affiliations:** 1 The First Affiliated Hospital of Shihezi University, Shihezi City, Xinjiang 832000 China; 2 Department of Gastroenterology, The First Affiliated Hospital of Shihezi University, No. 107, North Second Road, Shihezi City, Xinjiang 832000 China

**Keywords:** Esophageal Squamous Cell Carcinoma, Lipid metabolism, Prediction model, Machine learning algorithm, ACOT9

## Abstract

**Background:**

Esophageal squamous cell carcinoma (ESCC) is a major cause of cancer-related mortality worldwide, with a high prevalence and poor prognosis in specific regions. Despite advancements in clinical care, the identification of reliable biomarkers for accurate survival prediction remains a significant challenge, hindering personalized treatment.

**Methods:**

This study utilized genomic and clinical data from TCGA and GEO databases, applying ten machine learning algorithms to develop a prognostic model based on lipid metabolism-related genes. The Random Survival Forest (RSF) algorithm was identified as the optimal framework, supplemented by immune infiltration analysis and in vitro functional validation of the key gene ACOT9.

**Results:**

The resulting 33-gene signature achieved superior performance, with a C-index of 0.708 and survival prediction area under the curve (AUC) values approaching 1.0 in the training cohort. High-risk scores were significantly correlated with advanced histological grade, tumor stage, and increased regulatory T-cell infiltration. Furthermore, silencing of ACOT9 markedly suppressed the proliferation, migration, and invasion of ESCC cells.

**Conclusions:**

This study provides a robust framework for ESCC risk stratification and identifies ACOT9 as a critical oncogenic driver, offering a novel scientific basis for optimizing clinical management strategies.

**Supplementary Information:**

The online version contains supplementary material available at 10.1186/s12876-026-04908-0.

## Introduction

Esophageal cancer (EC) is the seventh most prevalent malignancy and the sixth leading cause of cancer-related mortality worldwide [1]. Owing to the subtle nature of early clinical manifestations, many patients are diagnosed at advanced stages, resulting in poor prognosis [2]. Esophageal squamous cell carcinoma (ESCC) is the predominant pathological subtype in China, and the overall 5-year survival rate remains suboptimal despite recent advances in diagnostic modalities and therapeutic interventions [3, 4]. Consequently, the identification of novel biomarkers and the development of stratified management protocols are imperative for enhancing the clinical outcomes of ESCC.

Lipids function as critical energy substrates and structural components, and undergo continuous metabolic flux during cellular homeostasis [5]. Tumor cells exhibit a significantly increased demand for lipids to support functions such as protein modification and membrane biosynthesis [6]. Accumulating evidence has confirmed that glucose and lipid metabolic reprogramming not only drives the malignant biological behavior of tumor cells, but also mediates extracellular matrix stiffness, oxidative stress, and chronic inflammatory responses through multiple signaling pathways, which in turn form a positive feedback loop to further aggravate metabolic disorders in the tumor microenvironment [7]. Consequently, the key molecules involved in lipid metabolism may represent promising new targets for cancer therapy. Investigating their molecular mechanisms is crucial for developing novel treatment strategies and enhancing their therapeutic efficacy.

In this study, we employed multiple machine-learning algorithms to identify a lipid metabolism-related gene signature and constructed a prognostic prediction model for ESCC. Upon validation across four independent cohorts, the model demonstrated robust performance in predicting patient survival and tumor staging. Specifically, we identified acyl-CoA thioesterase 9 (ACOT9) as the central component of this signature and validated its oncogenic role through in vitro experiments. Overall, this predictive model and the identification of ACOT9 hold potential as valuable resources for prognostic assessment and clinical decision-making in future clinical practice.

## Materials and methods

### Data acquisition and preparation

Gene expression profiles and corresponding clinical data for ESCC were downloaded from The Cancer Genome Atlas (TCGA) via the UCSC Xena database (https://xenabrowser.net). The GSE53622 and GSE53624 datasets were obtained from the Gene Expression Omnibus (GEO) database (https://www.ncbi.nlm.nih.gov/geo/). These datasets were then combined to create merged cohorts. Batch effects were corrected using limma and sva packages. A total of 340 samples with expression data for 14,047 genes were included in the final merged cohort. Gene sets related to “LIPID BIOSYNTHETIC PROCESS”, “LIPID CATABOLIC PROCESS”, and “LIPID METABOLIC PROCESS” were retrieved from the Molecular Signatures Database (MsigDB; https://www.gsea-msigdb.org/gsea/msigdb). After merging these gene sets and removing duplicates, 1,168 unique lipid metabolism-related genes were identified for subsequent analyses.

### Model development using machine learning algorithms

Ten classical machine learning algorithms were employed: Random Survival Forest (RSF), LASSO (Least Absolute Shrinkage and Selection Operator) regression, Gradient Boosting Machine (GBM), Survival Support Vector Machine (Survival-SVM), Supervised Principal Components (SuperPC), ridge regression, Cox partial least-squares regression (plsRcox), CoxBoost, Stepwise Cox regression, and an Elastic Net (Enet). The model construction process was implemented using the R packages randomForestSRC(v3.2.1), glmnet, gbm, survivalsvm, superpc, plsRcox, CoxBoost, and survival.

For the RSF algorithm, models were built by setting the number of trees (ntree = 1000), the minimum sample size for terminal nodes (nodesize = 5), and the splitting rule (splitrule = ‘logrank’). Variable importance was calculated (importance = T) to identify the key features. The CoxBoost algorithm optimized the number of boosting steps (stepno) and penalty parameters via cross-validation, integrating feature selection during modeling. The Enet algorithm involved a grid search for tuning the regularization parameter alpha (within the range [0.1, 0.9]), combined with feature selection for a secondary modeling step. Stepwise Cox regression performed feature selection based on directional criteria and was combined with other models (e.g., CoxBoost and GBM) to form ensemble models. The GBM algorithm was configured with an initial number of trees (n.trees = 10000), tree depth (interaction.depth = 3), and learning rate (shrinkage = 0.001), and was applied in conjunction with feature selection. The plsRcox algorithm utilized cross-validation to select the optimal number of components (nt = 10) and was used either independently or in combination with feature selection methods. The SuperPC algorithm was configured with a specified number of thresholds (n.threshold = 20) and principal components (n.components = 1) and was used jointly with RSF or StepCox for dimensionality reduction prior to modeling. The Survival-SVM algorithm used a kernel function parameter set to gamma.mu = 1 and was applied independently or in combination with feature selection.

The model performance was evaluated using the concordance index (C-index). The C-index was calculated for each model using the coxph function in the survival package. Statistical differences in the C-index between the models were assessed using the compareC function to compute p-values. The performance of all algorithms was compared across the training and validation sets based on their C-index values. The algorithm that yielded the highest C-index value was selected for subsequent analysis.

The TCGA-ESCC dataset, which has a larger sample size, served as a training cohort. The GSE53622 and GSE53624 datasets, along with the merged dataset (Merge), were used as independent validation cohorts.

All models were optimized using 10-fold cross-validation to minimize overfitting risks and ensure robust predictive performance.

### Model evaluation and validation

Patients were stratified into high- and low-risk groups based on an optimal cut-off value determined using the survival package. The performance of the model was evaluated using the Area Under the Receiver Operating Characteristic Curve (AUC), Kaplan-Meier survival curves, and time-dependent ROC analysis. Higher AUC values indicate better model performance and greater predictive power for disease outcomes. The model was trained and established using the TCGA-ESCC dataset and subsequently validated across three independent datasets: GSE53622, GSE53624, and the Merge cohort.

To comparatively analyze the predictive performance of our model, we searched PubMed for prognostic models for ESCC published from 2020 to 2023. Gene sets of published models were retrieved. The C-index of our model was then directly compared with that of existing models across all four cohorts: GSE53622, GSE53624, Merge, and TCGA.

### Functional enrichment analysis of the prognostic model

Gene Ontology (GO), Kyoto Encyclopedia of Genes and Genomes (KEGG), and reactome gene sets were obtained from MSigDB. Pearson’s correlation analysis was performed using genes included in the prognostic model. Genes with an absolute correlation coefficient |r| > 0.5 and *P* < 0.01 were selected, categorizing the top 50 positively and top 50 negatively correlated genes separately. Enrichment analysis of these gene sets was performed using the GSVA package to assess their involvement in specific biological processes and pathways.

### Analysis of immune cell infiltration between risk groups

The IOBR R package was used to evaluate immune cell infiltration levels using two distinct algorithms: EPIC and xCell. Differences in immune infiltration between the high- and low-risk groups were analyzed across TCGA-ESCC, GSE53622, and GSE53624 cohorts. The results were visualized as a heat map using the pheatmap package.

### Immunohistochemistry (IHC)

Twenty-five paraffin-embedded ESCC tissue specimens and fifteen normal esophageal tissues specimens archived between 2020 and 2024 at the Department of Pathology, The First Affiliated Hospital of Shihezi University, were included in this study.

The experimental procedure was as follows: Paraffin sections were deparaffinized and rehydrated. Antigen retrieval was performed at a high temperature and pressure for 8 min. Endogenous peroxidase activity was blocked by incubating sections with a peroxidase blocker for 10 min in the dark. The sections were then incubated overnight at 4 °C with a primary antibody against ACOT9 (diluted 1:200). The following day, the sections were rewarmed at 37 °C, incubated with a secondary antibody (diluted 1:500) at room temperature for 30 min, developed with DAB, counterstained with hematoxylin, and mounted with neutral gum.

IHC scoring was performed using the H-score method. The scoring criteria were as follows: staining intensity was graded as 0 (no visible staining), 1 (weakly positive), 2 (moderately positive), and 3 (strongly positive). The H-score was calculated as H-score = ∑(Pi × i), where Pi denotes the percentage of stained cells at each corresponding intensity score i, with a range of 0 to 300.

### Cell culture and cell Transfection

The ESCC cell lines EC109 and KYSE150 were acquired from the Saibaikang Biotechnology Company. Small interfering RNAs (siRNAs) were synthesized by GenePharma. EC109 and KYSE150 cell lines were seeded into 6-well plates and cultured in a 37 °C incubator with 5% CO₂ until reaching approximately 80% confluence. Three distinct siRNAs targeting ACOT9 (si-ACOT9-1, si-ACOT9-2, and si-ACOT9-3) were transfected into the experimental groups, and a non-targeting control siRNA (si-NC) was used as the negative control. Transfection efficiency was confirmed using western blot analysis.

### Cell counting Kit-8 (CCK-8) proliferation assay

Logarithmically growing cells were seeded in 96-well plates at a density of 1 × 10⁴ cells per well. CCK-8 solution (10% v/v, APEx-BIO, USA) was added to each well, followed by incubation at 37 °C for 2 h. Absorbance was measured at 450 nm using a microplate reader. The same measurement procedure was repeated 24, 48, 72, and 96 h after seeding.

### Colony formation assay

Logarithmically growing cells were seeded into 6-well plates at a density of 1000 cells/well and cultured for 14 days. The culture medium was then discarded. Colonies were fixed with 4% paraformaldehyde for 10 min, stained with 0.1% crystal violet for 5 min, gently rinsed with running water, air-dried, and photographed.

### Cell migration and invasion assays

Cell migration and invasion abilities were assessed using Transwell chambers. For the invasion assay, Matrigel was diluted 1:8 with pre-cooled DMEM, and 80 µL of the mixture was applied to the upper chamber of the insert and allowed to solidify. The chamber was left uncoated for the migration assay. Cells in the logarithmic growth phase were resuspended in serum-free DMEM at a density of 4 × 10⁴ cells/mL. Then, 200 µL of the cell suspension was added to the upper chamber (Matrigel-coated for invasion assay or uncoated for migration assay). The lower chamber was filled with 500 µL of DMEM supplemented with 20% fetal bovine serum as a chemoattractant. After 48 h of incubation at 37 °C, cells that had migrated or invaded through the membrane were fixed with 4% paraformaldehyde for 10 min, stained with 0.1% crystal violet for 5 min, and rinsed with water. Non-migrated or invading cells on the upper surface of the membrane were gently removed using a cotton swab. The membranes were air-dried, and the cells on the lower surface were counted under a microscope and photographed.

### Western blot

Total protein was extracted using RIPA lysis buffer supplemented with PMSF and a phosphatase inhibitor cocktail. The protein concentration was quantified using a BCA protein assay kit (Thermo Fisher Scientific). Protein samples were mixed with 1/4 volume of loading buffer, denatured by heating at 100 °C for 10 min, and separated by SDS-PAGE. Subsequently, the proteins were transferred onto polyvinylidene fluoride membranes (Millipore). The membrane was blocked with Rapid Blocking Buffer (New Cell & Molecular Biotech, Suzhou, China) at room temperature for 30 min and then incubated overnight at 4 °C with a primary antibody against ACOT9 (Wuhan SanYing Biotechnology). After washing, the membranes were incubated with horseradish peroxidase (HRP)-conjugated secondary antibody (anti-rabbit or anti-mouse) at room temperature for 1 h. Protein bands were visualized using an ECL detection reagent. GAPDH was used as an internal loading control. The band intensity was quantified using ImageJ software.

### Statistical analysis

Bioinformatics data processing, analysis, and visualization were performed using R software (v4.3.1), while additional statistical analyses were performed using SPSS software (v25.0). To compare two groups, Student’s t-test was used for normally distributed data, whereas the Mann-Whitney U test was used for non-parametric variables. Comparisons across multiple groups were conducted using ANOVA. A two-tailed *P* < 0.05 was considered to indicate statistical significance.

## Results

### Development and evaluation of the prognostic model

**Fig. 1:**
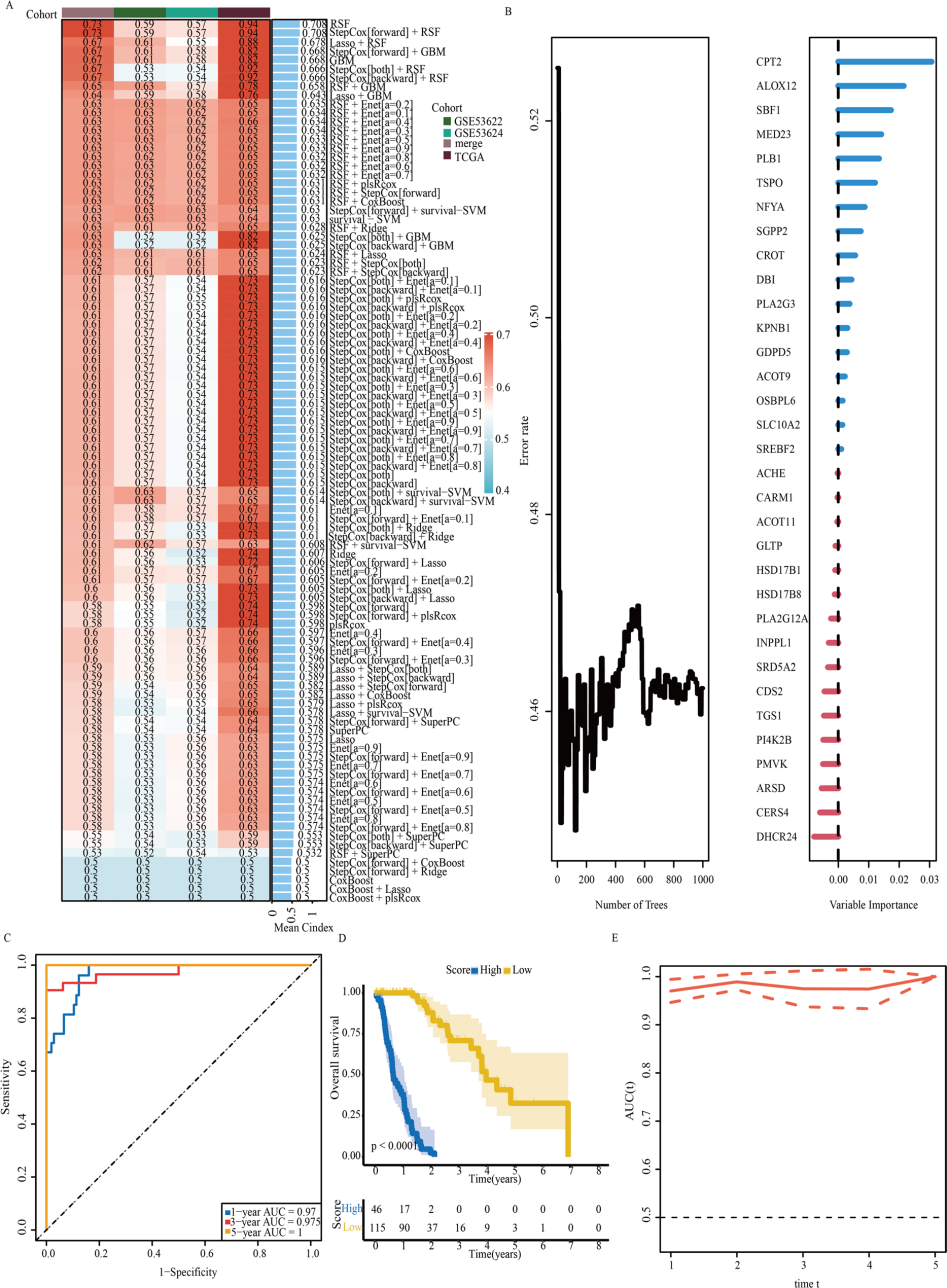
Construction and validation of the machine learning-based prognostic model. **A** Training and performance evaluation of multiple machine learning algorithms across four cohorts (GSE53622, GSE53624, Merge, and TCGA) based on C-index values. **B** Predictive performance of the RSF algorithm and identification of the 33-gene prognostic signature. **C** Assessment of model discriminatory capacity via ROC curves. **D** Kaplan-Meier survival analysis demonstrating prognostic stratification between high- and low-risk groups. **E** Evaluation of longitudinal model accuracy using time-dependent ROC curves

Machine learning algorithms and their combinations were applied to the four cohorts, with the model yielding the highest C-index, which was identified as the optimal model. Among these, the RSF algorithm demonstrated superior performance with a C-index of 0.708 (Fig. 1A). This RSF-based model was constructed using a signature of 33 genes (Fig. 1B). The performance of the resulting model was initially evaluated within the TCGA-ESCA training cohort. Notably, the AUC values for the 1-, 3-, and 5-year survival predictions were 0.97, 0.975, and 1.0, respectively (Fig. 1C). Kaplan-Meier analysis further revealed that patients in the high-risk group exhibited significantly worse overall survival compared to those in the low-risk group (*P* < 0.0001; Fig. 1D). Finally, time-dependent ROC analysis confirmed the robust discriminatory power of the model, with AUCs approaching 1.0 (Fig. 1E).

**Fig. 2:**
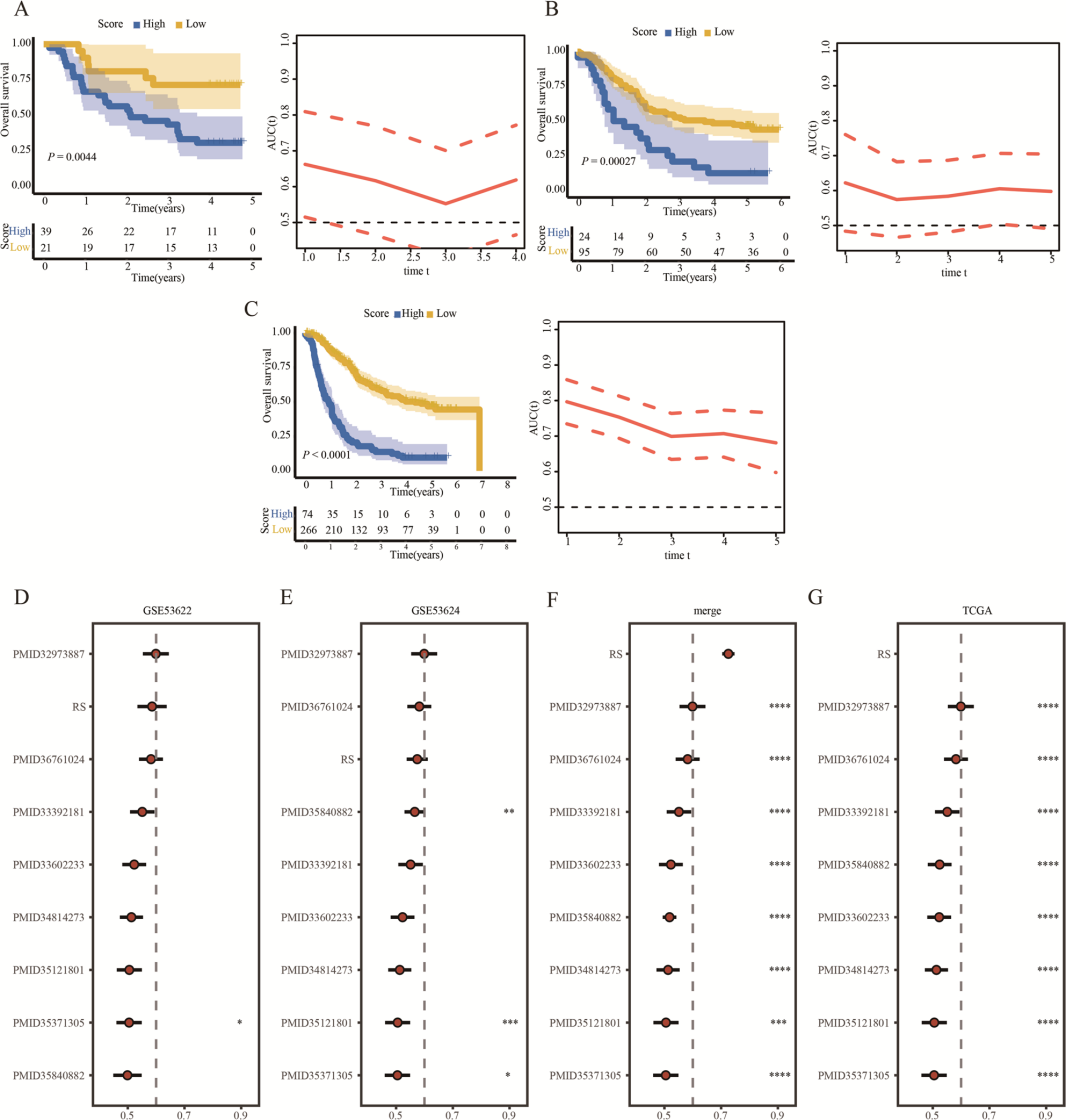
External validation and comparative benchmarking of the prognostic model. **A–C** External validation of the prognostic model across three independent cohorts (GSE53622, GSE53624, and the Merge cohort). Left panels: Kaplan-Meier survival curves depicting prognostic stratification between high-risk and low-risk groups. Right panels: Time-dependent ROC curves evaluating the longitudinal predictive accuracy of the model. **D–G** Comparative benchmarking of the developed model against established ESCC prognostic models. Predictive performance was assessed and compared across four cohorts (GSE53622, GSE53624, Merge, and TCGA) utilizing C-index values

To assess generalizability, the model was externally validated using the GSE53622, GSE53624, and merged cohorts. Receiver operating characteristic (ROC) analysis revealed that the AUC values across the validation cohorts spanned between 0.6 and 0.8. Kaplan-Meier analysis consistently demonstrated that patients in the high-risk group exhibited a significantly inferior prognosis relative to those in the low-risk group (*P* < 0.05; Fig. 2A-C). In the training cohort, the AUC reached 1.0, thereby raising the possibility of overfitting; however, sustained performance across independent validation cohorts confirmed the model’s robustness and absence of significant bias. Benchmarking against previously published prognostic models for ESCC demonstrated that our predictive model achieved superior performance. Specifically, the model’s C-index ranked second, third, first, and first in the GSE53622, GSE53624, Merge, and TCGA cohorts, respectively (Fig. 2D-G).

### Clinical relevance of the prognostic model

This study utilized an RSF-based prognostic model to evaluate the association between risk scores and clinicopathological features including tumor grade and stage.

**Fig. 3:**
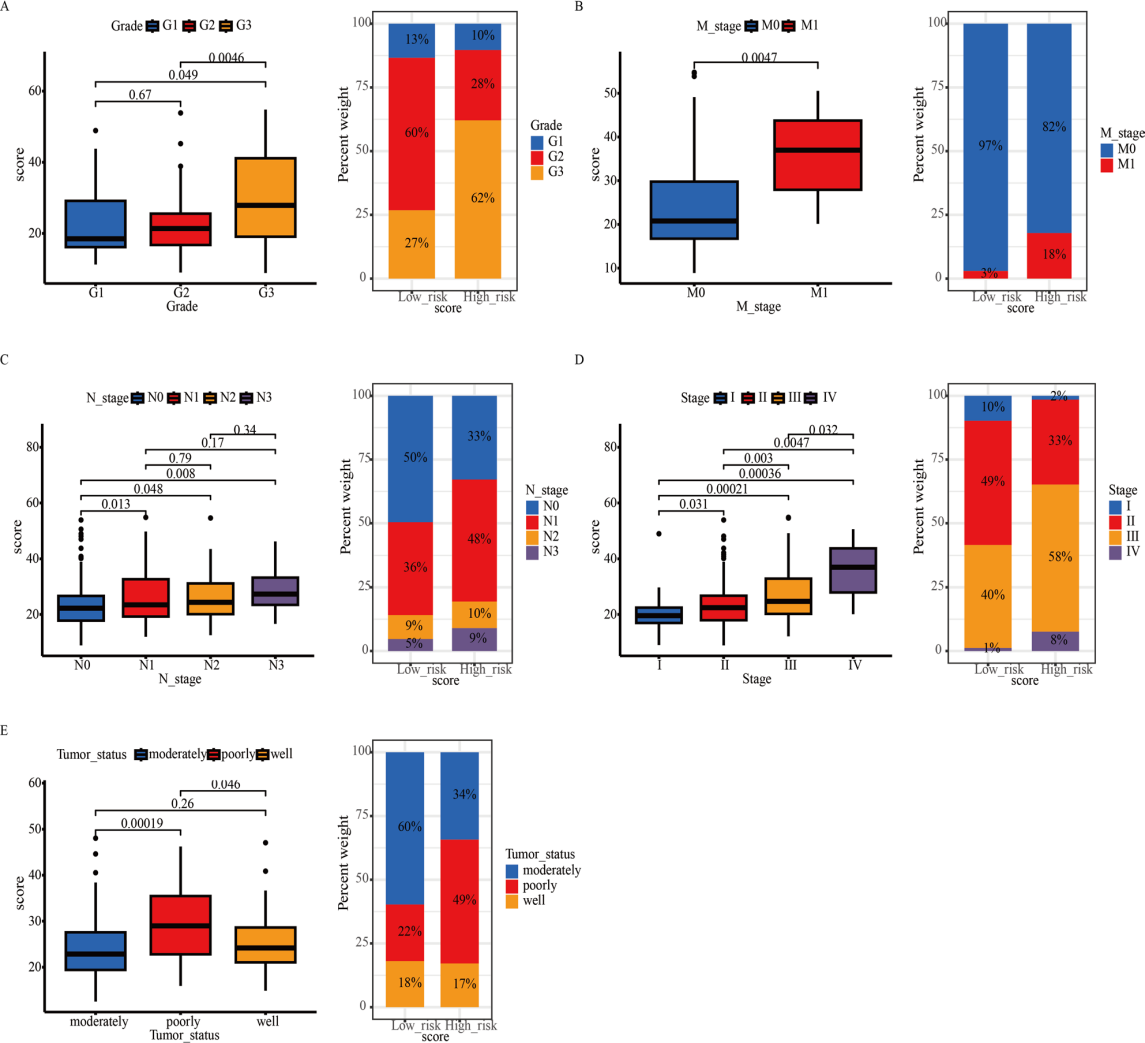
Association between the prognostic risk score and clinicopathological characteristics. **A** Left: Comparison of risk scores across histological grade groups. Right: Distribution of histological grades within high- and low-risk groups. **B** Left: Comparison of risk scores between M stage groups. Right: Distribution of M stage within high- and low-risk groups. **C** Left: Comparison of risk scores across N stage categories. Right: Distribution of nodal (N) stage within high- and low-risk groups. **D** Left: Comparison of risk scores across overall TNM stages. Right: Distribution of clinical stages within high- and low-risk groups. **E** Left: Comparison of risk scores across tumor differentiation status. Right: Distribution of differentiation status within high- and low-risk groups

The histological grade (G1: well-differentiated; G2: moderately differentiated; G3: poorly differentiated) reflects the degree of cellular differentiation and biological aggressiveness. Our findings demonstrated that the risk scores positively correlated with higher histological grades. Significant differences in risk scores were observed between G3 and G2 (*P* = 0.0046), and between G3 and G1 (*P* = 0.049). Furthermore, the prevalence of G3 tumors was markedly higher in the high-risk group (62%) than in the low-risk group (27%). These data suggest that high-risk patients tend to harbor tumors with lower differentiation and heightened aggressiveness (Fig. 3A).

Tumor staging is a clinical indicator of disease progression. The risk scores escalated with increasingly advanced disease stages. Specifically, risk scores were significantly higher in M1 cases than in M0 cases (*P* = 0.0047), concurrent with a higher frequency of M1 cases in the high-risk group (18% vs. 3%; Fig. 3B). Similarly, the risk scores for the N1, N2, and N3 stages were significantly higher than those for N0 (*P* = 0.013, *P* = 0.048, and *P* = 0.008, respectively), and the proportions of nodal involvement (N1–3) were consistently higher in the high-risk group than in the low-risk group (Fig. 3C). Regarding the overall stage, the risk score for stage IV was significantly higher than that for stages III (*P* = 0.032), II (*P* = 0.0047), and I (*P* = 0.00036). Additionally, the high-risk group had a higher proportion of stage IV (8%) and stage III (58%) patients than the low-risk group (stage IV: 1%, stage III: 40%; Fig. 3D).

The tumor differentiation status serves as an indicator of malignancy. The risk scores for the poorly differentiated group were significantly higher than those for the moderately (*P* = 0.046) and well-differentiated groups (*P* = 0.00019). Consistently, the proportion of poorly differentiated tumors was higher in the high-risk group (49%) than in the low-risk group (22%; Fig. 3E).

### Functional enrichment analysis of the prognostic model

To explore the potential biological functions and underlying mechanisms of the signature genes, Gene Set Enrichment Analysis (GSEA) was conducted. Following the Pearson correlation analysis, the 100 most significantly correlated genes (comprising the top 50 positive and 50 negative correlates) were selected for functional enrichment analysis using GO, KEGG, and Reactome pathways.

**Fig. 4:**
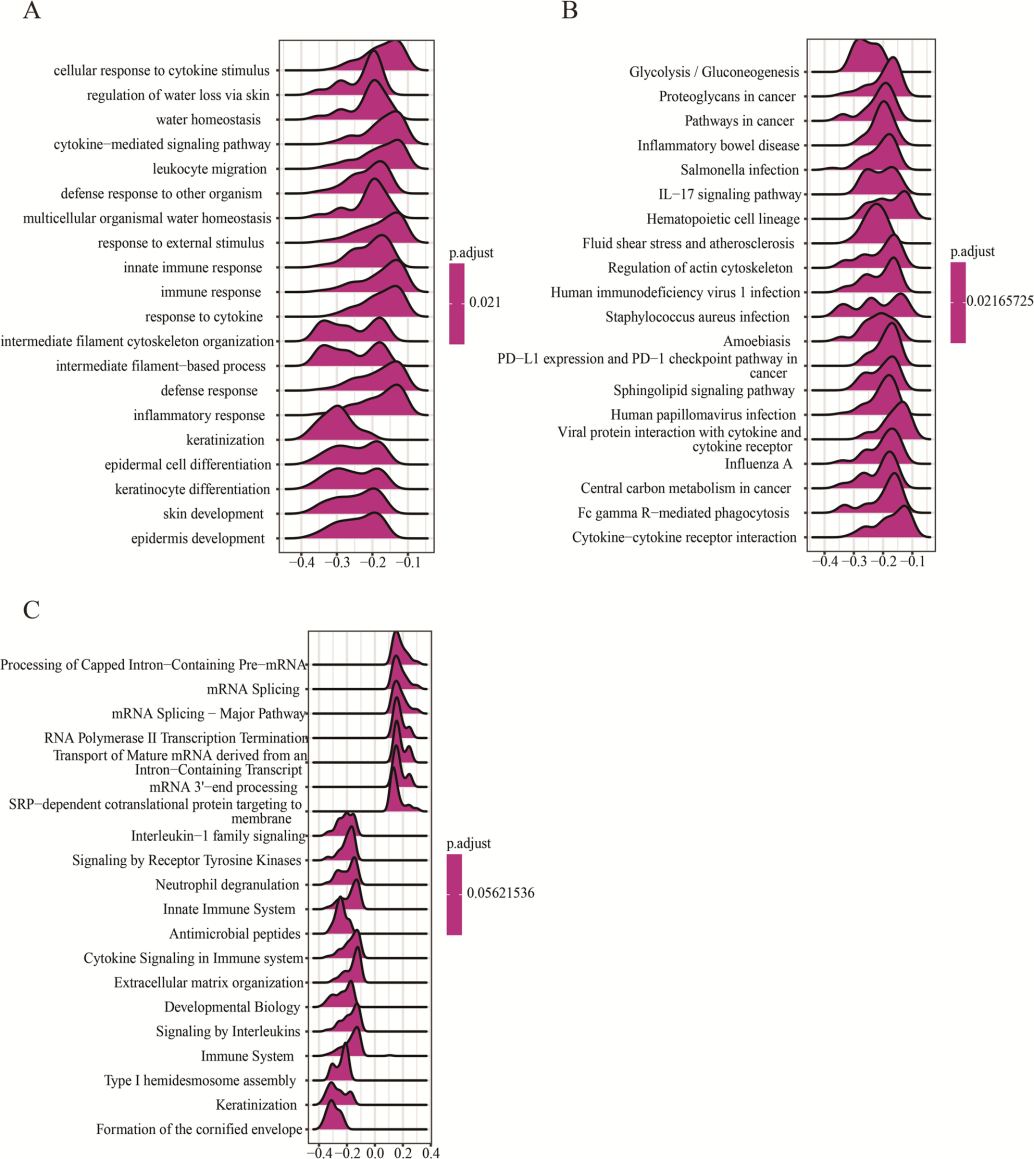
Functional enrichment analysis of signature-associated genes. **A** GO enrichment analysis of genes associated with the prognostic model. **B** KEGG pathway analysis of signature-associated genes. **C** Reactome pathway enrichment analysis of the signature-associated genes

GSEA targeting GO biological processes revealed significant negative enrichment (adjusted *P* < 0.05) in categories, including cellular response to cytokine stimulus, cytokine-mediated signaling, and various immune and inflammatory responses (Fig. 4A). KEGG pathway analysis further identified significant negative enrichment (adjusted *P* < 0.05) in pathways involved in cancer, IL-17 signaling, PD-L1/PD-1 checkpoint signaling, and sphingolipid metabolism (Fig. 4B). Reactome pathway analysis consistently highlighted the suppression of pathways associated with cytokine signaling, the innate immune system, and interleukin signaling (Fig. 4C).

### Immune cell infiltration analysis

**Fig. 5:**
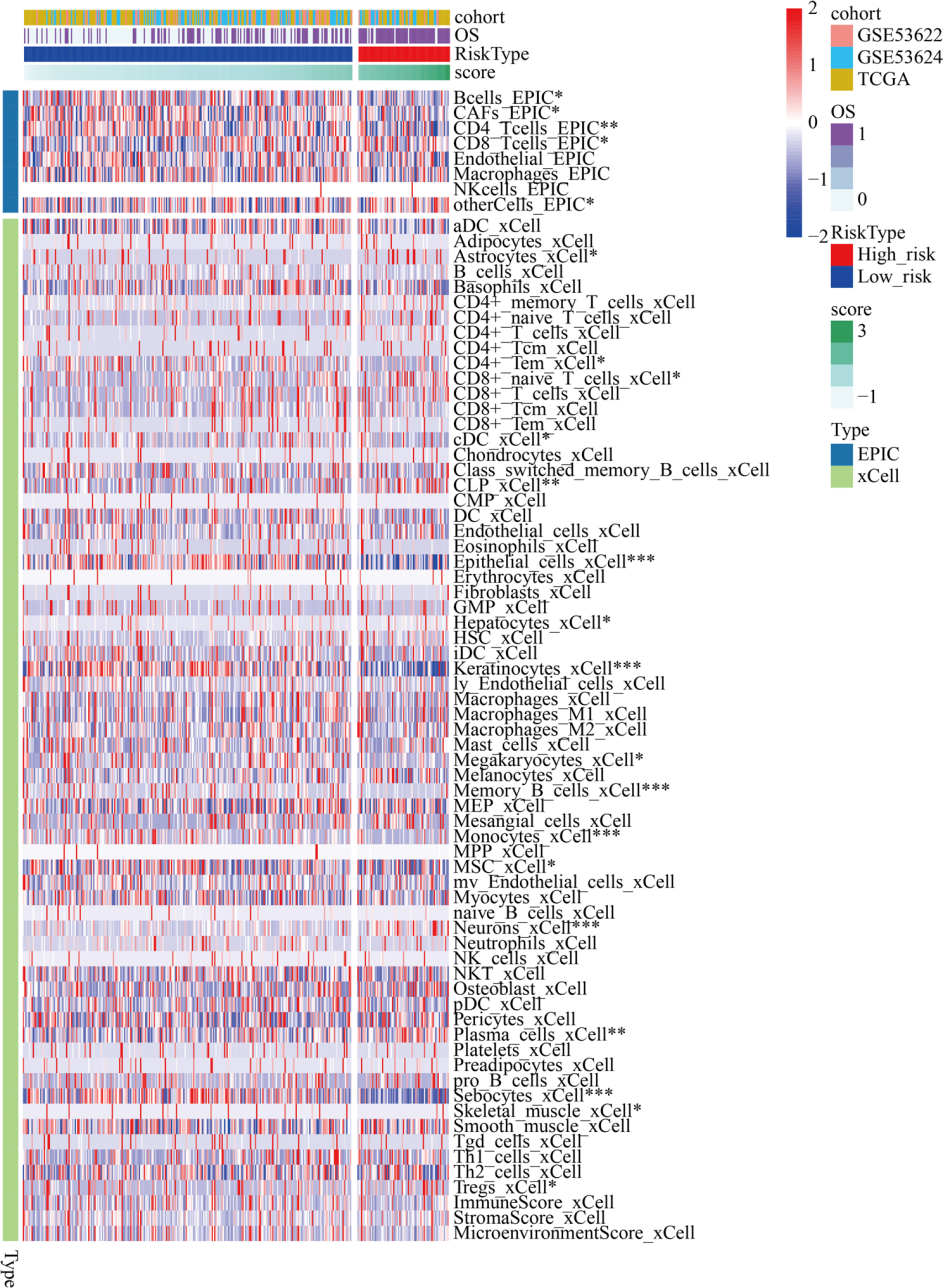
Comprehensive immune infiltration landscape stratified by risk score. The landscape of tumor immune infiltration is shown across three cohorts (TCGA, GSE53622, and GSE53624), with patients stratified by risk score. Utilizing multi-algorithm deconvolution (EPIC, and xCell), the heatmap depicts the relative abundance of diverse immune cell populations within the tumor microenvironment (TME) for the high- and low-risk groups

To further elucidate the immune landscape associated with the prognostic model, the tumor microenvironment (TME) was characterized across the risk groups. Our findings revealed that compared with the low-risk group, the high-risk group exhibited significantly enriched infiltration of CD8 + T cells, common lymphoid progenitors, memory B cells, and regulatory T cells (Tregs), along with enhanced tumor antigen presentation and effector cell activity (*P* < 0.05). Conversely, the infiltration levels of immune populations, such as B-lineage cells, CD4 + T cells, and dendritic cells, as well as stromal components, such as keratinocytes and epithelial cells, were significantly reduced in the high-risk group (all *P* < 0.05; Fig. 5).

### The impact of ACOT9 on malignant behaviors in ESCC cell lines

**Fig. 6:**
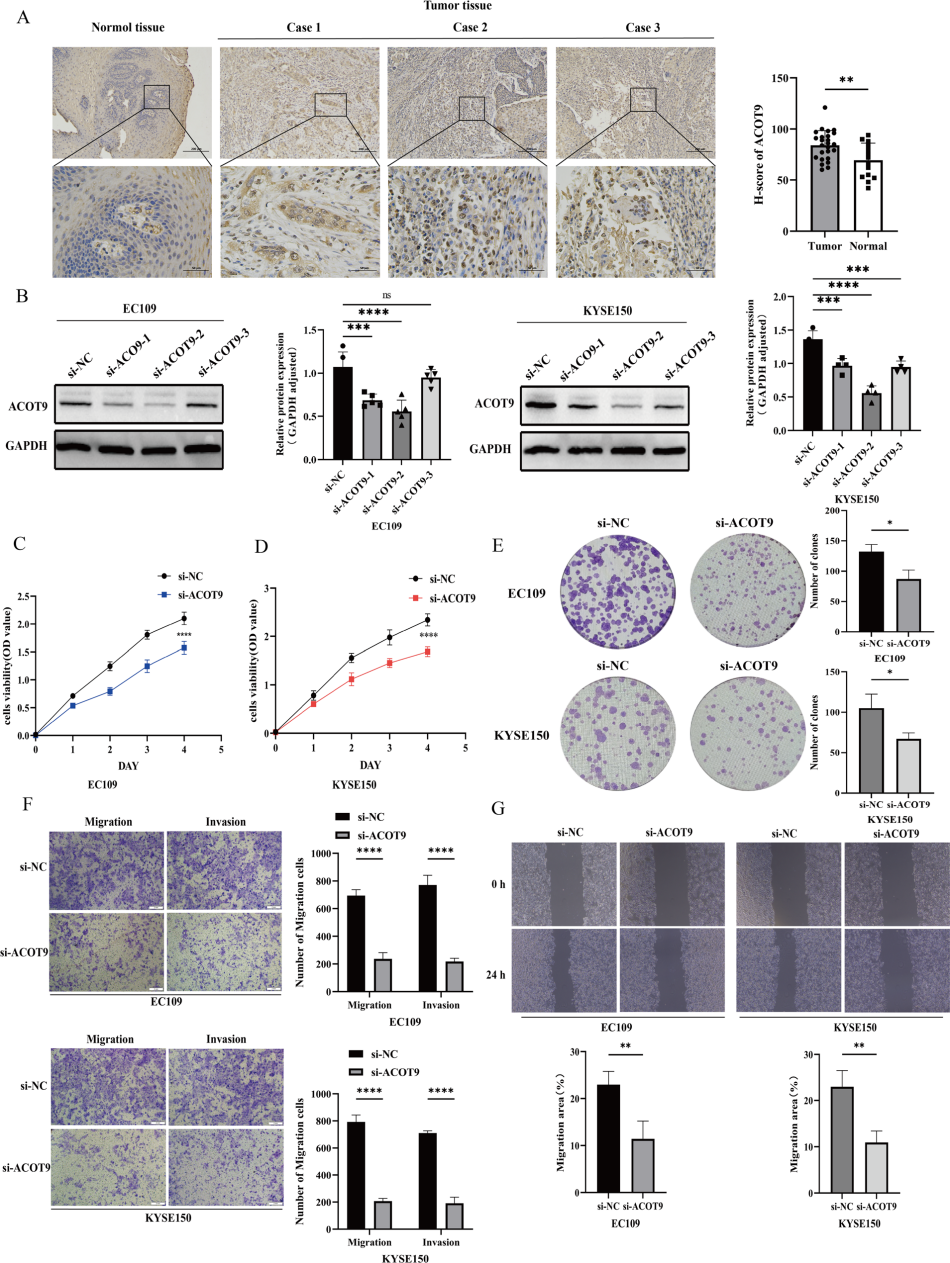
Experimental validation of the functional role of ACOT9 in ESCC cell lines. **A** Immunohistochemical (IHC) analysis of ACOT9 protein expression in ESCC tissues. **B** Validation of ACOT9 knockdown efficacy via Western blot. **C**,** D** Evaluation of ESCC cell proliferation using Cell Counting Kit-8 (CCK-8) assays. **E** Assessment of clonogenic capacity in ESCC cell lines via colony formation assays. **F** Quantification of migratory and invasive capacities using Transwell assays. **G** Wound-healing assays evaluating the motility of ESCC cell lines. **P < 0.01, ***P < 0.001, ****P < 0.0001

IHC was performed to evaluate ACOT9 protein expression levels in ESCC tissues. IHC analysis revealed that ACOT9 protein expression was significantly higher in 25 ESCC tissues than in 15 normal esophageal tissues (*P* = 0.006; Fig. 6A).

The knockdown efficacy of ACOT9 was assessed via western blotting. Among the three siRNAs tested, si-ACOT9-2 exhibited the most potent knockdown effect in both EC109 and KYSE150 cell lines relative to the si-NC control (*P* < 0.0001; Fig. 6B). Consequently, si-ACOT9-2 was selected for the subsequent functional experiments.

CCK-8 assays revealed that ACOT9 silencing significantly suppressed cell proliferation, as evidenced by the reduced absorbance at 450 nm compared to that in the si-NC group (*P* < 0.0001; Fig. 6C, D). Consistent with these findings, colony formation assays showed that ACOT9 knockdown markedly impaired the colony-forming capacity, with a significant reduction in colony size and number (*P* < 0.01; Fig. 6E).

Furthermore, Transwell assays demonstrated that the migratory and invasive capabilities of ESCC cells were significantly attenuated following ACOT9 knockdown (*P* < 0.0001; Fig. 6F), whereas wound healing assays showed that the wound closure rate was markedly retarded (*P* < 0.01; Fig. 6G).

## Discussion

In 2020, approximately 604,100 new cases of esophageal cancer and 544,076 related deaths were reported globally, nearly half of which occurred in China, representing a formidable public health challenge [1]. Although the 10-year survival rate for EC has improved from 20.9% to 30.3% in recent decades, the prognosis remains poor [8]. Clinical determinants such as insidious early symptoms, delayed diagnosis, and diminished sensitivity to chemoradiotherapy contribute to the poor outcomes observed in patients with ESCC. Although histopathology and TNM staging remain the conventional gold standards for predicting patient outcomes, these paradigms often lack the precision required for individualized prognostic forecasting. Therefore, clinicians require a reliable prognostic model to optimize diagnostic and therapeutic strategies. Lipid metabolism is pivotal for tumor progression because malignant cells undergo metabolic reprogramming to harness lipid-derived energy for rapid proliferation and metastasis. Thus, targeting of lipid metabolic pathways has emerged as a novel anti-cancer strategy [9]. Furthermore, the advent of machine learning, characterized by its capacity to analyze large-scale datasets and identify biologically relevant genes, has revolutionized prognostic modeling [9, 10]. While prior studies have established lipid metabolism-based prognostic signatures for ESCC [11], these efforts have been constrained by three key limitations: reliance on a single algorithm (predominantly LASSO regression), limited external validation in only one independent cohort, and the near absence of experimental validation to confirm the oncogenic roles of signature genes. To address these critical methodological and translational gaps, we systematically evaluated ten distinct machine learning algorithms across four independent patient cohorts to develop a prognostic signature based on lipid metabolism-related genes, which provides a more reliable foundation for personalized clinical decision-making in ESCC.

Using a lipid metabolism-related gene set, we evaluated various machine learning algorithms to construct a prognostic model, ultimately identifying the RSF algorithm that yielded the highest C-index as the optimal framework. Systematic multi-algorithm evaluation has also been widely recommended for the construction of polygenic risk models for complex diseases, because it can minimize the impact of data heterogeneity and improve the cross-population generalizability of the model [12]. Through training and validation across multiple datasets, the model demonstrated AUC values of 0.97, 0.975, and 1.0 for 1-, 3-, and 5-year survival predictions, respectively. Time-dependent ROC curve analysis revealed an AUC approaching unity, demonstrating the model’s high specificity and robust discriminatory capacity for long-term survival. Concurrently, the results from three independent validation cohorts consistently demonstrated that high-risk patients exhibited an inferior prognosis compared to those in the low-risk group, with AUCs ranging from 0.6 to 0.8. Although the AUC in the training set approached 1.0, stable performance across three independent external cohorts (AUCs 0.6–0.8) combined with multi-layered overfitting mitigation strategies confirms no substantial overfitting; the high training set AUC reflects the model’s ability to capture core lipid metabolism patterns in ESCC. Comparative analysis with established ESCC prognostic models further indicated that the predictive performance of our model was superior to that of existing frameworks. The RSF-based prognostic model effectively stratified patients, revealing significant differences in tumor grade, stage, and histological differentiation between the high- and low-risk groups. High-risk patients exhibited elevated tumor grades, which are associated with reduced cellular differentiation and increased biological aggressiveness, thereby providing a biological rationale for their inferior prognosis. Furthermore, the proportions of advanced-stage disease and distant metastasis were significantly increased in the high-risk group, further validating the prognostic utility of the model. By leveraging these predictive features, clinicians can enhance the accuracy of prognostic assessments in clinical practice, thereby facilitating the development of tailored therapeutic plans and management strategies. In conclusion, this model not only provides a novel theoretical basis for prognostic assessment in ESCC but also serves as a valuable tool for future clinical practice.

The TME is pivotal in modulating cancer progression and therapeutic efficacy. Beyond malignant cells, the TME comprises a complex assembly of immune and inflammatory cells, fibroblasts, adipocytes, signaling molecules, and extracellular matrix. Within this milieu, immune cells orchestrate tumor growth, metastasis, and immune escape through the interplay of activating and suppressive signaling mechanisms. Functional enrichment analyses indicated that the signature genes likely drive disease progression by downregulating immune-related pathways. Characterization of the immune landscape across the risk groups revealed that the high-risk phenotype was associated with diminished infiltration of CD4^+^ T cells, B cells, and dendritic cells, juxtaposed with the enrichment of CD8^+^ T cells and regulatory T cells (Tregs). This finding is consistent with recent studies showing that abnormal lipid metabolism in tumor cells can reshape the TME, leading to the depletion of anti-tumor effector immune cells and enrichment of immunosuppressive cell populations, which are key mechanisms of tumor immune escape and immunotherapy resistance [13]. CD4^+^ T cells are essential mediators of anti-tumor immunity [14], and their functional suppression may facilitate a phenotypic shift toward an immunosuppressive TME [15]. While CD8^+^ T cells are crucial effector cells in anti-tumor immunity capable of recognizing and eliminating tumor cells via cytotoxicity [15], their absolute count in high-risk patients, although increased, is likely offset by functional exhaustion due to Treg-mediated suppression. This results in a state of high cellular density but impaired functional competence [16]. The establishment of this immunosuppressive niche may be a primary determinant of poor prognosis in high-risk groups. Additionally, the diminished presence of B cells and dendritic cells may further impair antigen presentation and humoral immune responses, thereby exacerbating immune escape [17]. Collectively, the remodeled immune composition within the TME represents a critical driver of poor prognosis [18], aligning with the functional insights inferred from our model and reinforcing its predictive accuracy. These findings provide a robust theoretical basis for targeted interventions, such as Treg-directed therapies, which have significant therapeutic potential in high-risk patients. Machine learning-based TME subtyping can more accurately screen patient populations that can benefit from immunomodulatory therapy, providing a basis for the development of individualized immunotherapy regimens for ESCC [19].

To validate the expression of key signature genes, we retrospectively analyzed our previous transcriptomic microarray data from Kazakh ESCC tissues. The results indicated that ACOT9 and PLB1 were significantly upregulated (*P* < 0.05), whereas ALOX12 and DBI were significantly downregulated (*P* < 0.05) [20]. Consequently, ACOT9 was selected for subsequent functional validation. Previous studies established that ACOT9 promotes oncogenesis and metastasis in hepatocellular carcinoma by modulating lipid metabolic reprogramming, thereby serving as a potential therapeutic target [21]. Our findings confirmed the overexpression of ACOT9 in ESCC tissue samples. Following the establishment of ACOT9-knockdown ESCC cell lines, CCK-8 and colony formation assays revealed that ACOT9 silencing significantly impaired the proliferative and clonogenic capacities. Furthermore, Transwell and wound healing assays demonstrated that ACOT9 knockdown attenuated the migratory and invasive potential of these cells. Collectively, these findings suggest that ACOT9 plays an oncogenic role and represents a viable therapeutic target for ESCC. Nevertheless, further studies involving a broader repertoire of cell lines and in *vivo* animal models are warranted to fully elucidate the effects on lipid metabolism and the specific molecular mechanisms involved.

In conclusion, we developed a novel prognostic model for ESCC based on lipid metabolism-related genes, leveraging an innovative multi-algorithm integration strategy and rigorous cross-cohort validation. The model demonstrated moderate predictive accuracy for both patient survival and tumor staging. By elucidating the underlying biological mechanisms and performing experimental validation of the key gene ACOT9, this study provides novel mechanistic insights that may contribute to a potential foundation for individualized diagnosis and management of ESCC.

## Supplementary Information

Supplementary Material 1.

Supplementary Material 2.

## Data Availability

Gene expression profiles and corresponding clinical data for ESCC were downloaded from The Cancer Genome Atlas (TCGA) via the UCSC Xena database (https://xenabrowser.net). The GSE53622 and GSE53624 datasets were obtained from the Gene Expression Omnibus (GEO) database (https://www.ncbi.nlm.nih.gov/geo/). Gene sets related to “LIPID BIOSYNTHETIC PROCESS”, “LIPID CATABOLIC PROCESS”, and “LIPID METABOLIC PROCESS” were retrieved from the Molecular Signatures Database (MsigDB; https://www.gsea-msigdb.org/gsea/msigdb).

## Abbreviations

ESCC: Esophageal squamous cell carcinoma
TCGA: The Cancer Genome Atlas
GEO: Gene Expression Omnibus
RSF: Random Survival Forest
AUC: Area under the curve
TME: Tumor microenvironment
IHC: Immunohistochemistry
CCK-8: Cell Counting Kit-8
GO: Gene Ontology
KEGG: Kyoto Encyclopedia of Genes and Genomes
