# Supplementary material for: Establishment of prognostic prediction model based on lipid metabolism related genes in esophageal squamous cell carcinoma by machine learning algorithms
Source: BMC Gastroenterol. 2026 May 12;26:409. doi: 10.1186/s12876-026-04908-0 (PMC13335382; doi:10.1186/s12876-026-04908-0)
Supplement: Supplementary file 1 — Supplementary Material 1. [file 12876_2026_4908_MOESM1_ESM.pdf]

editage

Editing Certificate

Issued On April 03, 2026

This document certifies that the manuscript listed below has been edited to ensure language and grammar accuracy and is error free in these aspects. The edit was performed by professional editors at Editage, a brand of Cactus Communications. The author's core research ideas were not altered in any way during the editing process. The quality of the edit has been guaranteed, with the assumption that our suggested changes have been accepted and the text has not been further altered without the knowledge of our editors.

QUALITY GUARANTEE • QUALITY GUARANTEE • QUALITY GUARANTEE
THE editage PROMISE

Manuscript Title

Establishment of a prognostic prediction model based on lipid metabolism related genes in esophageal squamous cell carcinoma using machine learning algorithms

Authors

Peng Wang, Zhenyuan Feng, Weigang Chen

EIIDQ_2

Job Code

Prabh Grewal, Senior Vice President, Editage

VERIFY AT
https://editage.cn/ec
verify

VERIFICATION CODE
EC-260406-
20RCXW
