# Supplementary material for: Establishment of prognostic prediction model based on lipid metabolism related genes in esophageal squamous cell carcinoma by machine learning algorithms
Source: BMC Gastroenterol. 2026 May 12;26:409. doi: 10.1186/s12876-026-04908-0 (PMC13335382; doi:10.1186/s12876-026-04908-0)
Supplement: Supplementary file 2 — Supplementary Material 2. [file 12876_2026_4908_MOESM2_ESM.pdf]

Thermo Fisher maker #26616

KYSE-150:

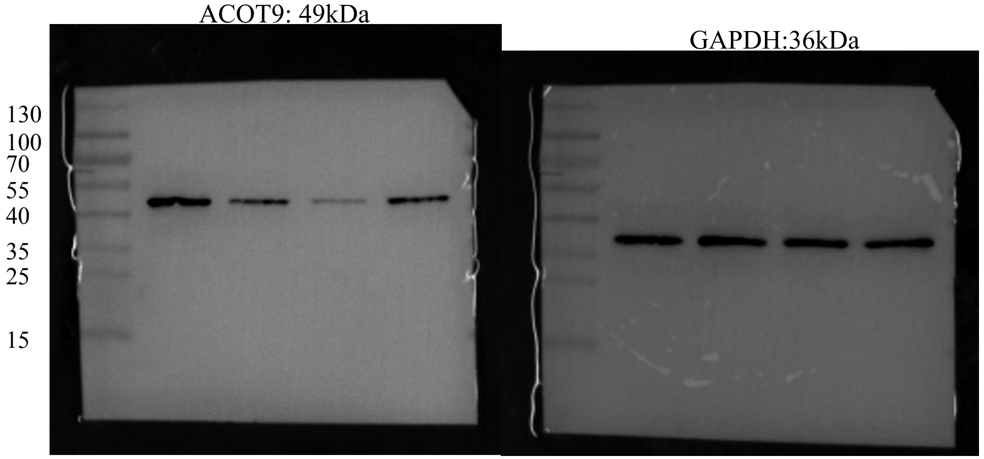

The membrane was stripped with stripping buffer after ACOT9 immunoblotting, then re-incubated with GAPDH antibody.

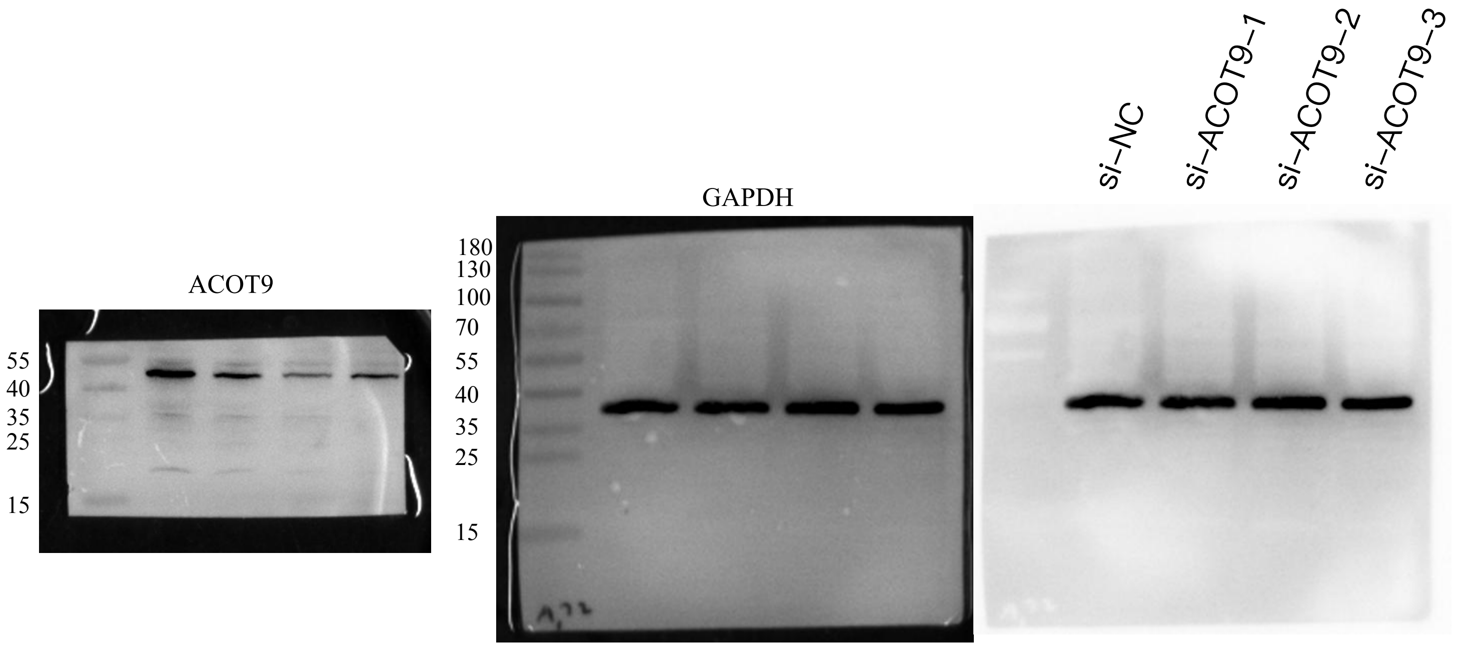

The original immunoblotting figure of the article.

EC-109:

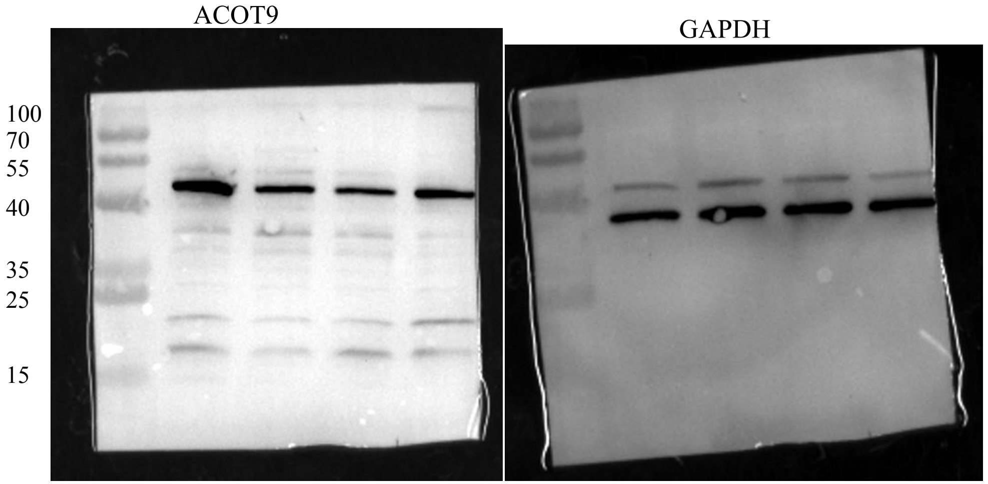

The membrane was stripped with stripping buffer after ACOT9 immunoblotting, then re-incubated with GAPDH antibody.

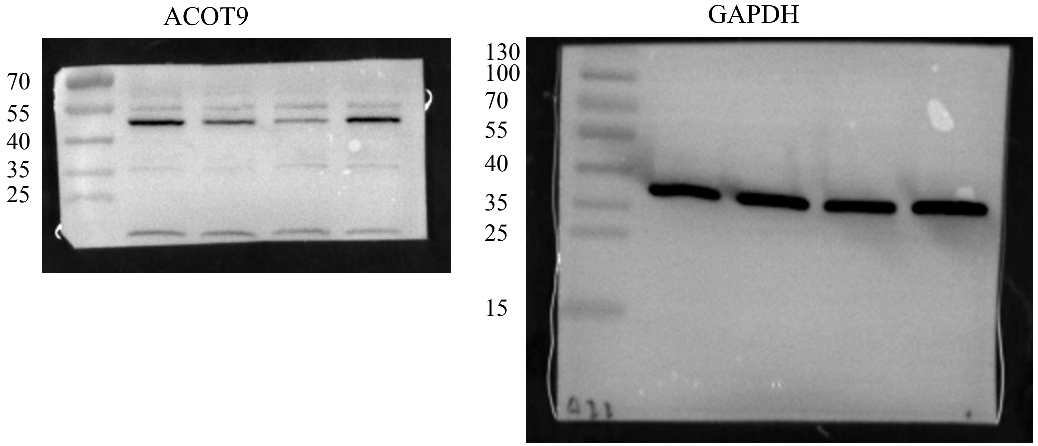

The original immunoblotting figure of the article.
